# Food Addiction and Its Impact on Weight-Based Stigma and the Treatment of Obese Individuals in the U.S. and Australia

**DOI:** 10.3390/nu6115312

**Published:** 2014-11-21

**Authors:** Natalia M. Lee, Wayne D. Hall, Jayne Lucke, Cynthia Forlini, Adrian Carter

**Affiliations:** 1 School of Population Health, the University of Queensland, Herston, QLD 4029, Australia; E-Mail: natalia.evans@uq.net.au; 2 Centre for Youth Substance Abuse, the University of Queensland, Herston, QLD 4029, Australia; E-Mail: w.hall@uq.edu.au; 3 Australian Research Centre in Sex, Health and Society, Faculty of Health Sciences, La Trobe University, Melbourne, VIC 3000, Australia; E-Mail: j.lucke@latrobe.edu.au; 4 UQ Centre for Clinical Research, the University of Queensland, Herston, QLD 4029, Australia; E-Mail: c.forlini@uq.edu.au; 5 School of Psychological Sciences, Monash University, Melbourne, VIC 3181, Australia

**Keywords:** addiction, attitudes, obesity, stigma, responsibility

## Abstract

It is argued that food addiction explanations of obesity may reduce the significant stigma levelled at obese and overweight individuals. We surveyed 479 adults to determine the prevalence of food addiction in the U.S. (*n* = 215) and, for the first time, in Australia (*n* = 264) using the Yale Food Addiction Scale (YFAS). We also assessed the level of weight-based stigma in this population. The prevalence of food addiction in our Australian sample was 11%, similar to U.S. participants and consistent with previous studies. Those who met criteria for diagnosis had a larger mean BMI (33.8 kg/m^2^) than those who did not (26.5 kg/m^2^). Overall, the level of stigma towards others was low and differed significantly based on BMI, predominately among normal weight and obese participants (*p* = 0.0036). Obese individuals scored higher on certain measures of stigma, possibly reflecting individual experiences of stigma rather than negative attitudes towards other obese individuals (*p* = 0.0091). Despite significant support for a “food addiction” explanation of obesity, participants still valued personal responsibility in overcoming obesity and did not support coercive approaches to treat their “addiction”.

## 1. Introduction

Neurobiological research on overeating in animals and humans [1,2,3] has identified many of the mechanisms and dysregulated neural pathways that are involved in overconsumption and satiety. An addiction model of obesity has been proposed in which both the neurobiological and behavioural mechanisms of overeating mirror those operating in substance dependence based on the Diagnostic and Statistical Manual of Mental Disorders, 4th edition (DSM-IV) criteria [2]. Despite this, fundamental disagreement exists within the scientific community on how to define what has been referred to as food addiction or whether such a psychiatric diagnostic category is justified or helpful [4]. Modifications to the 5th edition of the Diagnostic and Statistical Manual of Mental Disorders (DSM-5) raise additional questions to food addiction’s relevance with the newly defined “substance-related and addictive disorders”. The inclusion of gambling as an addictive disorder within the DSM-5 does broaden the category to potentially include other behaviours in future editions. While the core components of food addiction (*i.e.*, substance taken in a larger amount or for a longer period than intended, persistent efforts to cut down or control use, craving and substance use continued despite affiliated problems) still strongly resemble those of substance-related and addictive disorders [5], additional research is needed to assess how well food addiction resembles substance-related and addictive disorders or non-substance-related behavioural disorders based on the DSM-5. While significant advancements have been made in the understanding of food addiction via animal models and behavioural and neuroimaging studies in humans, the development and application of an addiction model of obesity is still largely nascent.

The Yale Food Addiction Scale (YFAS) is the primary method of assessing and diagnosing food addiction [6]. It has been used to study the prevalence of food addiction in various U.S. populations, such as undergraduates [6] and individuals with binge eating disorder [7], as well as in other international samples [8,9]. It has recently been paired with actual food stimuli and a dopamine uptake inhibitor to directly measure appetitive processes [10]. While Pedram and colleagues [9] report a positive association between food addiction diagnosis and body mass index (BMI), a brief review conducted by Meule [8] demonstrates a non-linear relationship, whereby rates of food addiction are higher among under-, as well as over-weight individuals, compared with normal weight counterparts, that increases further with obesity.

Greater acceptance of the claim that obesity is a form of food addiction may have important implications for the way that obesity is treated. Paradoxically, we found previously that support for the concept of food addiction was associated with the belief that obese individuals are largely responsible for their weight [11]. Excess weight is also a highly stigmatized condition, whereby responsibility for weight and weight gain is placed on the individual [12], often at the expense of contributing factors, such as genetics, health conditions and obesogenic environments. Instantiating obesity as the result of a compulsive “brain disorder” or food addiction could discourage obese individuals from engaging in healthy lifestyle behaviours and foster an overreliance on pharmacotherapy for weight loss. Equating obesity with food addiction could even justify the use of coercive treatments if obese individuals are seen to suffer from a form of addiction over which they have limited control. Brain-based explanations of under- and over-eating have been used to justify invasive neurosurgical treatments, such as deep brain stimulation [13,14], as well as the need for more paternalistic coercive interventions for the good of the patient [15]. It is therefore important to consider what impact an addiction model of obesity may have on weight-based stigma.

The prevalence of food addiction has been assessed in individual countries, but no single study has yet examined food addiction across countries. It is also unclear what effect the prevalence and acceptance of food addiction may have on weight-based stigma. North America and Australia possess the highest BMI among developed countries [16]. We found significant differences in the aetiology of addiction in these countries [11], which may provide insights on the impact of neurobiological understandings of obesity and stigma. The aims of this study were to: (1) determine and compare the prevalence of individuals meeting the criteria for food addiction as measured by the YFAS in samples of U.S. and Australian residents; (2) assess whether levels of weight-based stigma varied with BMI or food addiction diagnosis; and (3) compare responses based on country of residence in two Westernized countries with high population rates of obesity.

## 2. Experimental Section

### 2.1. Sample Recruitment

This study was conducted using a sample of U.S. and Australian residents 18 years and older primarily recruited through online advertising with supplementary recruitment from an online staff newsletter at the University of Queensland and snowball sampling [11]. This study was approved by a Health Research Ethics Committee at the University of Queensland.

### 2.2. Survey Measures

The online survey involved a series of multiple-choice questions (using 5-point Likert scales) to assess the levels of weight-based stigma held by members of the public toward obese individuals. The survey also examined public attitudes towards the causes and risk factors for obesity, treatment endorsement and the impact of food addiction [11].

#### 2.2.1. Measures of Food Addiction and Eating Disorders

The presence of food addiction and eating disorders were identified using the YFAS and the Eating Disorder Examination Questionnaire (EDE-Q), respectively. The YFAS is based on the DSM-IV’s substance dependence criteria and is the only diagnostic tool for food addiction displaying both internal reliability and convergent validity [6].

The EDE-Q examines restraint over eating, eating concern, shape concern and weight concern over the past 28 days [17]. Twenty-two of the EDE-Q’s 28 questions required for score calculation were used in the present study. The frequency of the responses provided by the 7-point scales ranges from 0 (if a feature is not present) to 6 (if a feature is present every day).

#### 2.2.2. Stigmatisation and Discrimination

Participants were asked to answer a series of questions assessing the level of stigmatization towards a fictional character in the following vignette:

Sarah is 5′3″ (161 cm) and weighs 200 pounds (91 kg) at 30 years of age. She has tried, unsuccessfully, to lose weight on multiple occasions. Doctors have told Sarah that she is obese and have expressed concerns about her health.

Questions were adapted to fit the vignette’s central theme of obesity and to measure weight-based stigma from previous research on the stigmatisation of mental illnesses found in the Attitudes to Mental Illness Questionnaire (AMIQ) and the 1996 and 2006 General Social Survey (GSS) (see Table 1) [18,19]. The GSS is a stratified multistage probability sample survey conducted in the U.S., and the AMIQ is a validated instrument used in various medical and mental health stigma research [18,19]. Response codes ranged from −2 to +2 for individual items, signifying either stigma or lack of it, respectively. Individual item codes were summed to yield a total stigma score ranging from −10 to +10. Responses to the questions measuring treatment outcomes were analysed separately.

Participants were also asked about the main cause of the character’s obesity in the vignette, with response options: “biological causes”, “environment”, “genetics or family history”, “personal choice” and “other”. Consistent with our previous analysis examining beliefs about the cause of obesity in general [11], “biological causes” and “genetics or family history” were combined during analysis, as the two represent causes of obesity external to personal control. To assess the impact of the vignette on perceived causes of obesity, responses were compared to beliefs about the causes of obesity published previously [11].

**Table 1:** Questions used to measure levels of stigma based on the vignette.

Stigmatisation and Discrimination
I would be comfortable if Sarah was my colleague at work.
*Strongly agree^+2^/Agree^+1^/Don’t know^0^/Disagree^−1^/Strongly disagree^−2^*
I would be comfortable inviting Sarah to a dinner party.
*Strongly agree^+2^/Agree^+1^/Don’t know^0^/Disagree^−1^/Strongly disagree^−2^*
I would be comfortable having Sarah as an in-law.
*Strongly agree^+2^/Agree^+1^/Don’t know^0^/Disagree^−1^/Strongly disagree^−2^*
How likely do you think it would be for Sarah’s husband to leave her?
*Very likely^−2^/Quite likely^−1^/Don’t know^0^/Unlikely^+1^/Very unlikely^+2^*
How likely do you think it would be for Sarah to get fired?
*Very likely^−2^/Quite likely^−1^/Don’t know^0^/Unlikely^+1^/Very unlikely^+2^*

#### 2.2.3. Demographic and Weight Information

Participants were asked to report their age, sex, ethnicity, family and personal history of selected medical conditions, highest level of completed education, annual household income, country of residence and height and weight (to determine BMI).

### 2.3. Statistical Methods

Means and standard deviations were computed for the continuous variables of interest and standardized proportions computed for all variables of interest. Chi-squared analyses were calculated using the statistical software R (R Foundation for Statistical Computing, Vienna, Austria).

## 3. Results

### 3.1. Sample Characteristics

A total of 610 individuals began the online survey with a completion rate of 79%, yielding a final sample of 479 participants comprised of 215 adults from the U.S. and 264 from Australia (see Table 2). Detailed analyses of the sample’s characteristics have been reported previously [11]. The U.S. sample varied from national averages for sex, age, race, education and income, but closely reflected the sociodemographic characteristics of the current U.S. population for weight and median age [20]. The Australian sample varied from national averages for sex, age, race, education, income and weight [21,22], but closely reflected the current Australian nationwide obesity prevalence and median age [21,22]. The U.S. and Australian samples were similar to one another.

**Table 2:** Sample Characteristics (*n* = 479).

Sample Characteristics	*n* (%)
**Sex**	
Female	383 (80)
Male	93 (19)
**Age**	
18–24	73 (15)
25–34	154 (32)
35–44	87 (18)
45–54	82 (17)
55–64	59 (12)
65–84	24 (5)
**Race**	
Caucasian	389 (81)
Indigenous	31 (6)
Asian	17 (4)
Hispanic	11 (2)
African American	9 (2)
Other	8 (2)
**Self-reported Body Mass Index (BMI)**	
Underweight, BMI <18.5 kg/m^2^	14 (3)
Normal weight, BMI 18.5–24.9 kg/m^2^	228 (48)
Overweight, BMI 25–29.9 kg/m^2^	104 (22)
Obese, BMI >30 kg/m^2^	133 (28)
**Education**	
High school	75 (16)
2-Year vocational/technical degree	33 (7)
College graduate	166 (35)
Postgraduate degree	204 (43)
**Household Income (U.S. Dollars)**	
<$25,000	51 (11)
$25,000–49,999	86 (18)
$50,000–74,999	84 (18)
$75,000–99,999	71 (15)
$100,000+	187 (39)
**Family History of Health Condition**	
Alcoholism	167 (35)
Anorexia nervosa	19 (4)
Binge eating disorder	40 (8)
Bulimia nervosa	25 (5)
Compulsive behaviours	112 (23)
Depression	257 (54)
Heavy tobacco use	229 (48)
Obesity	198 (41)
**Individual History of Health Condition**	
Alcoholism	18 (4)
Anorexia nervosa	24 (5)
Binge eating disorder	43 (9)
Bulimia nervosa	18 (4)
Compulsive behaviours	54 (11)
depression	173 (36)
Regular tobacco use	121 (25)
Obesity	146 (30)
**Country of Residence**	
Australia	264 (55)
U.S.	215 (45)

### 3.2. Prevalence of Food Addiction and Eating Disorders

The diagnosis of a food addiction was made using the Yale Food Addiction Scale (YFAS) [6], while the presence of an eating disorder was made using the Eating Disorder Examination Questionnaire (EDE-Q) [17]. The majority of participants (86%) reported having a persistent desire or unsuccessful attempts to cut down on eating certain foods, and 29% of participants continued to consume certain foods despite either psychological or physical problems arising from such food use. Twelve percent of respondents met the YFAS criteria for food addiction, and 13% of all participants demonstrated clinically significant impairment from food use (see Table 3). This proportion was not significantly different from prevalence rates (11.6%) found in a study of 353 undergraduates used to validate the YFAS [6]. The average food addicted individual was obese (mean (M) BMI = 33.8; SD = 10.8), while undiagnosed participants were on average overweight (M BMI = 26.5; SD = 7.3). The prevalence of food addiction did not vary significantly between the Australian and U.S. samples (X^2^(1) = 1.595, *p* = 0.207).

**Table 3:** Diagnosis of food addiction based on criteria and body mass index (BMI). DSM-IV, Diagnostic and Statistical Manual of Mental Disorders, 4th edition; YFAS, Yale Food Addiction Scale.

DSM-IV Diagnostic Criteria for Substance Dependence as Measured by the YFAS	Underweight *n* (%)	Normal Weight *n* (%)	Overweight *n* (%)	Obese *n* (%)	Total (*n* = 479) *n* (%)
Diagnosis of food dependence	1 (7)	10 (4)	16 (15)	32 (24)	59 (12)
Tolerance	1 (7)	25 (11)	19 (18)	45 (34)	90 (19)
Withdrawal	0 (0)	14 (6)	20 (19)	33 (25)	67 (14)
Substance take in larger amounts or for a longer-than-intended period	2 (14)	15 (7)	19 (18)	29 (22)	65 (14)
Persistent desire or unsuccessful efforts to cut down or control use	10 (71)	184 (81)	96 (92)	123 (92)	413 (86)
Large amount of time spent to obtain, use or recover	2 (14)	21 (9)	21 (20)	42 (32)	86 (18)
Social, occupational or recreational activities neglected or reduced due to use	0 (0)	14 (6)	14 (13)	24 (18)	52 (11)
Continued use despite “recurrent physical or psychological problem caused or exacerbated by the substance”	0 (0)	37 (16)	31 (30)	72 (54)	140 (29)
Clinically significant impairment	2 (14)	10 (4)	16 (15)	34 (26)	62 (13)
**Total**	14	228	104	133	

Participants’ responses to the EDE-Q were similar to data from Fairburn and Beglin’s [23] community-based sample of 243 young women (M = 1.55, SD = 1.21). Our sample yielded an overall mean score of 1.72 (SD = 0.96) based on the average of the four subscale scores: restraint (M = 1.64, SD = 1.14), eating concern (M = 0.81, SD = 0.81), shape concern (M = 2.38, SD = 0.81) and weight concern (M = 2.04, SD = 0.96). The overall EDE-Q score was higher among the 59 participants diagnosed with food addiction by the YFAS (M = 3.66, SD = 1.13) than those not so diagnosed (M = 1.44, SD = 1.08). In addition, there was a positive association between mean overall EDE-Q score and BMI: underweight (M = 1.23, SD = 1.50), normal weight (M = 1.13, SD = 1.01), overweight (M = 2.02, SD = 1.23) and obese (M = 2.54, SD = 1.30).

### 3.3. Stigmatisation of Obesity

**Figure 1:**
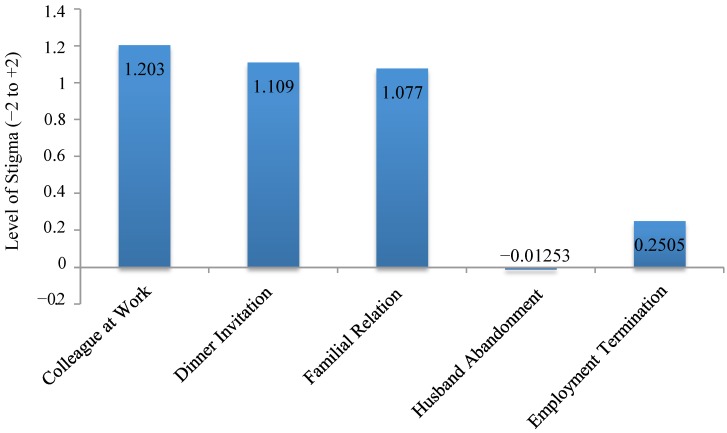
Levels of stigma elicited based on the vignette on a −2 to +2 scale for each individual item.

Stigma was measured using a vignette approach and the Attitudes to Mental Illness Questionnaire (AMIQ) [18], a validated measure of stigma in mental illness. The average level of stigma across the entire study sample was 3.63 (SD = 2.94), where +10 indicates the absence of stigma and −10 maximal stigma (see Figure 1). There was no difference in levels of stigma elicited in response to the vignette based on country of residence (*t* (477) = 1.241, *p* = 0.215) or diagnosis of food addiction (*t* (197) = 0.748, *p* = 0.455).

Total stigma varied significantly with BMI (*F*(3, 475) = 3.84, *p* = 0.0098), differing between those of normal weight and obese participants (*p* = 0.0036), with obese participants displaying the least amount of stigma (see Table 4). Normal weight participants were more likely (M = −0.11, SD = 0.74) than obese participants (M = 0.16, SD = 0.90) to believe that the character’s husband would leave her (*F*(3, 475) = 3.41, *p* = 0.0076). In contrast, obese participants supported employment termination (M = 0.05, SD = 1.02) more than normal weight counterparts (M = 0.35, SD = 0.84) (*F*(3, 475) = 3.44, *p* = 0.0091). A large proportion of participants were unsure whether the individual portrayed would be likely to get a divorce (57%) or get fired (45%).

**Figure 2:**
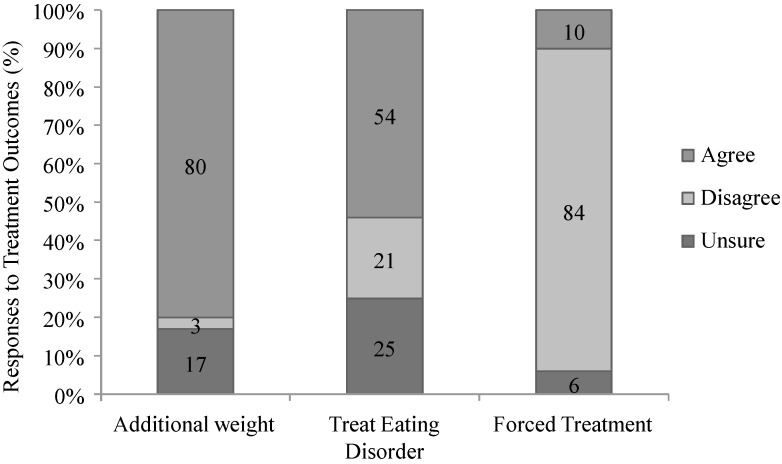
Attitudes toward obesity treatment based on the vignette.

In treatment outcomes, 80% of all participants thought that Sarah, the character in the vignette, was likely to gain additional weight; 54% supported treatment for an eating disorder, and 84% were against coerced weight-loss treatment (see Figure 2). There were no differences in the perceived likelihood of weight gain (X^2^(2) = 2.296, *p* = 0.317) nor in views on coerced treatment (X^2^(2) = 1.323, *p* = 0.516) based on country of residence. However, a significantly larger proportion of Australian participants favoured the treatment of an eating disorder than their American counterparts (X^2^(2) = 12.923, *p* = 0.002).

**Table 4:** Level of stigma and perceived treatment outcomes based on body mass index (BMI) and food addiction diagnosis.

	Total Stigma	Likelihood of Gaining Weight *n* (%)	Treatment for Eating Disorder *n* (%)	Forced Treatment *n* (%)
**BMI**				
Underweight	3.43	12 (86)	10 (71)	3 (21)
Normal weight	3.24	181 (79)	125 (55)	26 (11)
Overweight	3.62	87 (84)	68 (65)	10 (10)
Obese	4.32	105 (79)	56 (42)	8 (6)
**Diagnosis of Food Addiction**				
Diagnosed	3.45	116 (89)	74 (57)	13 (10)
Undiagnosed	3.69	269 (77)	185 (53)	34 (10)

Support for treating obesity as an eating disorder varied with BMI (X^2^(6) = 17.808, *p* = 0.007), with obese participants showing the least support. Participants who met the diagnosis of food addiction were more likely than those who did not to predict additional weight gain (X^2^(2) = 11.235, *p* = 0.004). No other differences were observed based upon a diagnosis of food addiction.

### 3.4. Causes of Obesity

When asked to assess the main cause of the character’s obesity as described in the vignette, 29% suggested a biological or genetic influence, 25% suggested personal choice and 18% were unsure about the cause(s). Views on the causes of obesity did not vary significantly by country of residence (X^2^(2) = 0.554, *p* = 0.758).

There was a statistically significant difference in views on the causality of obesity by BMI (X^2^(4) = 33.963, *p* < 0.001). Biological and genetic causes of obesity were more frequently endorsed by obese participants (M = 31.2, SD = 9.8), environmental causes by overweight participants (M = 26.2, SD = 6.1) and personal choice by normal weight participants (M = 24.9, SD = 6.1). This pattern was similar among normal and obese participants. Among overweight participants, personal choice (26%), followed by biological and genetic causes (23%) of obesity were frequently endorsed based on the vignette. Overweight participants were more likely to agree that overeating causes obesity than were obese participants (X^2^(3) = 3.941, *p* = 0.047).

There were no significant differences between those who did and did not meet criteria for a food addiction diagnosis in responses to questions about the causes of Sarah’s obesity. There was, however, a statistically significant difference between food addiction diagnosis and views on the general cause of obesity (X^2^(3) = 8.016, *p* = 0.046). Individuals with a food addiction were more likely to attribute obesity to external factors (*i.e.*, biology or genetics) rather than personal choice. Thirty five percent of individuals diagnosed with food addiction endorsed biological or genetic influences, followed by personal choice (27%), whereas undiagnosed counterparts predominately attributed obesity to personal choice (34%).

## 4. Discussion

### 4.1. Prevalence of Food Addiction

We found that the prevalence of food addiction was similar in both Australian and U.S. populations (12%) and was consistent with previous findings of a U.S. sample used in the validation of the YFAS [6]. In a sample of 652 Canadian adults, Pedram and colleagues [9] reported a prevalence of approximately 5%, significantly lower than in our sample and in a previous U.S. sample [6]. These differences could be due to marked variations in sample characteristics and study design. Future studies should assess the prevalence of food addiction in larger, representative samples of the general population utilizing the DSM-5’s substance-related and addictive disorders criteria and employing a revised YFAS.

In accordance with previous studies measuring the prevalence of food addiction [9,24], the risk for food addiction was positively associated with BMI. A diagnosis of food addiction was also more common among underweight participants than normal weight respondents, as shown previously in three studies of the prevalence of food addiction (*n* = 1499) [8]. This finding may suggest similarities with the presence of abnormal eating, as seen in anorexia nervosa or bulimia nervosa, and a food addiction diagnosis. Underweight individuals frequently reported persistent desire to cut down or control use, as well as taking problem foods in a larger amount (or for a longer duration) than intended and devoting considerable time to obtaining, using or recovering from eating this food. Some individuals who meet criteria for food addiction may also retain a lower BMI by engaging in compensatory behaviours, such as purging, exercise or dietary restrictions [8].

The mean EDE-Q score showed a positive association with BMI among normal weight and obese participants. This supports previous research in showing that both binge eating and the prevalence of binge eating disorder increase with BMI [25]. A higher mean EDE-Q score among the 59 participants diagnosed with food addiction is consistent with results from a study conducted by Gearhardt and colleagues [7] in which 57% of obese individuals with binge eating disorder also met criteria for a diagnosis for food addiction. In addition, a higher EDE-Q score among underweight participants, as compared with normal weight counterparts, may be indicative of an increased risk for disordered eating, such as anorexia nervosa and bulimia nervosa. The YFAS and EDE-Q’s similar results based on observed trends in BMI might provide an avenue for future research examining similarities between the symptomology of food addiction and eating disorders.

### 4.2. Obesity Treatment

The majority of the sample, including those who met the criteria for food addiction, supported the concept of food addiction, as demonstrated previously [11]. Treating food addiction with the aim of lowering obesity could increase perceived helplessness for weight-loss. A lack of support of coerced weight loss treatment based on the vignette in this study is consistent with our previous findings demonstrating greater support for programs that maximise control over eating, such as psychotherapy and educational programs [11]. Our sample was largely in favour of treatment and preventive measures that were elective, rather than coercive. We have previously shown widespread support (over 80%) for a food addiction model of obesity in this population [11]. Contrary to those that suggest a food addiction model of obesity may undermine responsibility in treatment, participants were strongly opposed to coerced weight-loss treatment and valued treatment approaches that maximised personal choice and responsibility for weight-gain. Further research should investigate participants’ understanding of the effects of treating obesity as a food addiction, particularly on stigma, and how this in turn affects public approval of treating obesity as an addiction.

### 4.3. Stigma

Stigma can have serious adverse psychological and social impacts on individuals with psychiatric disorders that significantly impair treatment outcomes [18,26]. Previous studies have suggested that high levels of weight-based discrimination in the general population parallel that of racial discrimination in the U.S. [27,28,29]. However, our study found relatively low levels of stigma in both Australian and American participants. Future research using stigma measures specific to obesity, such as the Antifat Attitudes Questionnaire [30], is needed to confirm whether our results reflect the views of the wider population or were the result of the specific tool used here. Future research could also employ a more detailed vignette, including the use of visual aids, in order to identify any stigmatising beliefs held by research participants.

In addition to yielding low levels of stigma, our vignette was not structured so as to distinguish between the different dimensions of stigma, such as social distance, sympathy and concern or anger and disgust. DePierre and colleagues [31] compared stigma associated with a food addiction diagnosis to that of other addictions, mental illness and physical disabilities and found that food addiction, while perceived similarly to obesity, generated greater social distance. While this study demonstrated that food addiction is viewed more favourably than other addictions [31], it did not employ a character-specific vignette. Further studies are needed to explore the different dimensions of stigma associated with food addiction while employing a variety of vignette characters.

It has been suggested that addiction models of disorders may reduce the stigma experienced by affected individuals [32]. An addiction model of obesity has been proposed to help elucidate the complex processes driving excess weight, as well as to improve treatment outcomes [33]. However, we found previously in this sample that support of an addiction model of obesity was not associated with altered perceptions of obese individuals or the treatment of obesity [11]. Similarly, a diagnosis of food addiction did not reduce stigma.

This is consistent with findings on attitudes towards psychiatric disorders. Pescosolido and colleagues [19] demonstrated that stigma was unchanged and possibly worsened despite increased public acceptance and endorsement of neurobiological explanations of depression and alcohol dependence. This is further supported by the observation that conditions under voluntary control are more likely to be stigmatized, especially when obesity is viewed as largely the result of personal choice [28].

There were marked discrepancies in perceptions based on BMI, particularly between normal weight and obese participants. The increased stigma among obese participants toward the likelihood of employed termination may reflect their greater experiences of work-based discrimination [12,34]. Previous large-scale studies demonstrate weight-based discrimination in the form of being passed over for a promotion or even an employment opportunity, as well as termination [12]. In addition, normal weight participants were more likely than obese participants to predict abandonment by a husband. This is consistent with research demonstrating weight-based discrimination directed at obese women by romantic partners and close family and friends [12].

As our sample was predominately female, it is unlikely that it accurately reflects the views of the population as a whole. Our sample of female participants yielded a total stigma score of 3.75 (SD = 2.97), while male counterparts yielded a score of 3.08 (SD = 2.76). While not significantly different, these results indicate a need for future research to elucidate the role of gender on weight-based discrimination. Future research should utilize a representative sample and examine attitudes towards obese males and individuals from different ethnicities and age strata.

### 4.4. Cause of Obesity and Addiction

Although the U.S. population predominately holds obese individuals responsible for their weight [34], our sample endorsed biological or genetic factors as the primary cause of obesity for the character in the vignette. Vignette approaches are believed to elicit more realistic responses and to reflect actual behaviours when faced with a specific situation, as opposed to broader reflections about general cases. The attribution of biomedical causes as the predominant cause of Sarah’s obesity shows public support of causes outside of one’s control, such as a food addiction.

Participants meeting a diagnosis of food addiction were more likely to attribute obesity to external biomedical influences, compared with personal choice ascribed by undiagnosed counterparts. This is consistent with attribution theory that suggests we are more likely to attribute cause for negative personal characteristics to external causes. The clinical relevance of such externalising views and the impact of food addiction models of obesity need to be examined.

### 4.5. Limitations

The study employed a convenience sample that was not representative of the general populations in the U.S. or Australia. As such, our findings cannot be readily generalised to the entire populations of these two countries. Sample bias may arise from limiting participants to those who have access to the Internet and the resources to complete online surveys. This study also included a significantly greater proportion of female respondents, possibly due to the greater interest of women in weight-based social issues. Future research should include a more representative sample of the general population in both the U.S. and Australia using targeted recruitment strategies (e.g., quota sampling) for salient demographic characteristics (e.g., gender, age) and computer-assisted telephone interviewing (CATI).

## 5. Conclusions

This study confirms previous estimates of the prevalence of food addiction in the U.S. and shows a similar pattern in Australia. The relationship between meeting criteria for food addiction and eating disorders warrants further examination. Overall levels of weight-based stigma in response to the vignette were lower than expected, possibly due to limitations of the measures employed. Responses to treatment outcomes reflect observed trends in obesity treatment (*i.e.*, additional weight gain) irrespective of stigma. Most participants believed that obesity treatment should be elective, rather than compulsory. Future research is needed to assess the impact of an addiction model of obesity on treatment outcomes.
